# Invasive Group A Streptococcal Infection and Vaccine Implications, Auckland, New Zealand

**DOI:** 10.3201/eid1706.100804

**Published:** 2011-06

**Authors:** Atheer Safar, Diana Lennon, Joanna Stewart, Adrian Trenholme, Dragana Drinkovic, Briar Peat, Susan Taylor, Kerry Read, Sally Roberts, Lesley Voss

**Affiliations:** Author affiliations: Auckland City Hospital, Auckland, New Zealand (A. Safar, S. Roberts);; The University of Auckland, Auckland (D. Lennon, J. Stewart);; Kidz First Children’s Hospital/Middlemore Hospital, Auckland (A. Trenholme);; North Shore Hospital, Auckland (D. Drinkovic, K. Read);; The University of Auckland/Middlemore Hospital, Auckland (B. Peat);; Middlemore Hospital, Auckland (S. Taylor);; Starship Children’s Hospital, Auckland (L. Voss)

**Keywords:** Streptococcus pyogenes, emm types, toxic shock syndrome, invasive disease, bacteria, New Zealand, vaccines, research

## Abstract

We aimed to assess the effect of invasive group A streptococcal (GAS) infection and the potential effects of a multivalent GAS vaccine in New Zealand. During January 2005–December 2006, we conducted prospective population-based laboratory surveillance of Auckland residents admitted to all public hospitals with isolation of GAS from normally sterile sites. Using *emm* typing, we identified 225 persons with confirmed invasive GAS infection (median 53 years of age; range 0–97 years). Overall incidence was 8.1 cases per 100,00 persons per year (20.4/100,000/year for Maori and Pacific Islanders; 24.4/100,000/year for persons >65 years of age; 33/100,000/year for infants <1 year of age). Nearly half (49%) of all cases occurred in Auckland’s lowest socioeconomic quintile. Twenty-two persons died, for an overall case-fatality rate of 10% (63% for toxic shock syndrome). Seventy-four percent of patients who died had an underlying condition. To the population in our study, the proposed 26-valent vaccine would provide limited benefit.

During the 2 decades since recognition of streptococcal toxic shock syndrome (STSS), there have been many publications on invasive group A streptococcal (GAS) infections, some population-based (*1**–**4*). The spectrum of infection caused by *Streptococcus pyogenes* varies widely from invasive disease, such as bacteremia, sepsis, necrotizing fasciitis (NF), and STSS, to noninvasive infection, most commonly pharyngitis with suppurative complications, such as otitis media, and nonsuppurative complications, such as acute rheumatic fever (ARF) and acute glomerulonephritis (APSGN).

GAS infection causes a substantial number of illnesses and deaths, especially in the developing world, with ≈500,000 deaths worldwide annually, attributable mostly to ARF and its sequelae, rheumatic heart disease, and invasive infection (*5*). GAS disease and its sequelae, including GAS pharyngitis, have been well documented in New Zealand (*6**–**12*; http://dnmeds.otago.ac.nz/departments/womens/paediatrics/research/nzpsu/pdf/2008_report.pdf).

With renewed interest in GAS vaccines (*13*), understanding the complete spectrum of disease, including invasive GAS disease, in diverse populations is essential. The vaccine most completely studied is a 26-valent vaccine based on *emm* types and M subtypes collected across GAS diseases from the United States (*14*). We previously published a population-based approach of laboratory surveillance for invasive bacterial diseases in Auckland’s public hospitals where all persons with acute disease would be admitted (*8**,**15**–**18*). Using this approach, we demonstrate the effects of invasive GAS on the Auckland population to complement our knowledge of other GAS-associated diseases by using prospectively collected incidence data, clinical characteristics, associated underlying conditions, and the associated *emm* types. This study also provided an opportunity to establish the direction of further investigations and to focus interventions in New Zealand.

## Methods

### Surveillance

We enrolled patients during January 1, 2005–December 31, 2006. Patients were included if they resided in metropolitan Auckland and had a GAS isolate cultured from a previously sterile body cavity. Patients with STSS were included in accordance with the consensus definition (*19*); STSS also was the diagnosis for patients who were dead on arrival or who died within 48 hours after illness onset and for whom laboratory data were insufficient in accordance with the methodology of Davies et al. (*1*). NF was defined as tissue necrosis diagnosed by histopathologic examination or by the treating surgeon during surgical debridement. Patients could have had >1 diagnosis, with the exception of bacteremia without a source. Clinical syndromes, such as skin or soft tissue infection, had to be accompanied by recovery of an isolate from a normally sterile site or specimen, such as blood, to meet the case definition. Nosocomial infection was defined as GAS infection in patients who had been hospitalized for >72 hours. Invasive GAS infection was defined as postpartum if it occurred in a woman who was pregnant or <30 days after delivery or who had clinician-defined puerperal fever, chorioamnionitis, or a septic abortion. Women from whom GAS was isolated from amniotic fluid or placenta alone were excluded (*20*). Our study was approved by the regional ethics committee and each hospital’s research committee and Maori research committee.

Data were collected from the microbiology laboratories serving all 3 Auckland regional District Health Board (DHB) hospitals, i.e., Auckland City Hospital and Starship Children’s Hospital (Auckland DHB); Middlemore Hospital, which includes Kidz First Children’s Hospital (Counties Manukau DHB); and North Shore Hospital and Waitakere Hospital (Waitemata DHB). All Auckland residents with serious medical illness would attend 1 of these hospitals.

Auckland (2006 population: 1,387,780), New Zealand’s largest city, comprises one third of the country’s population and is the country’s most ethnically diverse city. In 2006, 19.0% of residents self-identified as Asian, 14.4% as Pacific Islander, 11.1% as indigenous Maori, and 56.5% as European. The climate is temperate, with summer occurring during December through March. We used New Zealand birth data for infants <1 year of age and customized New Zealand census charts for DHBs as denominators.

We obtained demographic and clinical features by reviewing medical charts and electronic documents. To ensure complete surveillance, we requested International Classification of Diseases, 10th Revision, diagnoses from DHB data managers. We contacted the regional coroner and forensic pathologist to seek out records of deaths (including deaths in the community) caused by GAS infection and scrutinized intensive care unit (ICU) data for diagnoses of shock from GAS, STSS, or NF. Disease severity was determined by length of stay, ICU admission, and use of surgical and medical procedures.

We assigned each invasive GAS infection in the Auckland region a deprivation score by using the New Zealand Deprivation Index 2006 (www.moh.govt.nz). This index measures socioeconomic status (SES) in small areas according to 9 variables (income, income assistance, education, access to a car and phone, household crowding, employment, single-parent family, housing rented or owned).

### Laboratory Techniques

β-hemolytic colonies on blood agar were typed as Lancefield group A by using commercially available latex agglutination kits (Pro-Lab Diagnostics, Austin, TX, USA). Group A isolates were sent to Environmental Science and Research Laboratory (Wellington, New Zealand) for *emm* typing by using established procedures (*21*). Concordance between *emm* types and M serotypes has been established (*21*). Antimicrobial drug sensitivities were determined by routine methods (*22*).

### Estimates of Vaccine Benefit

We used *emm* typing to estimate the proportion of cases and deaths caused by *emm* types in the proposed 26-valent vaccine. These *emm* GAS types are 1, 2, 3, 5, 6, 11, 12, 13, 14, 18, 19, 22, 24, 28, 29, 33, 43, 59, 75, 76, 77, 89, 92, 94, 101, and 114 (*14*). We then calculated potential vaccine efficacy in the most at-risk Auckland populations: persons <5 years of age and >65 years of age.

## Results

### Epidemiology

During the 24-month study period, we identified 333 patients who potentially had invasive GAS infections. Of these, we excluded 118 who did not fulfill the inclusion criteria. The most common reasons for exclusion were isolation from a nonsterile site or residence outside metropolitan Auckland at the time of diagnosis. Using the electronic discharge summaries based on International Classification of Diseases, 10th Revision, coding, we identified and included 10 (4%) additional cases that fit the case definition.

**Figure 1:**
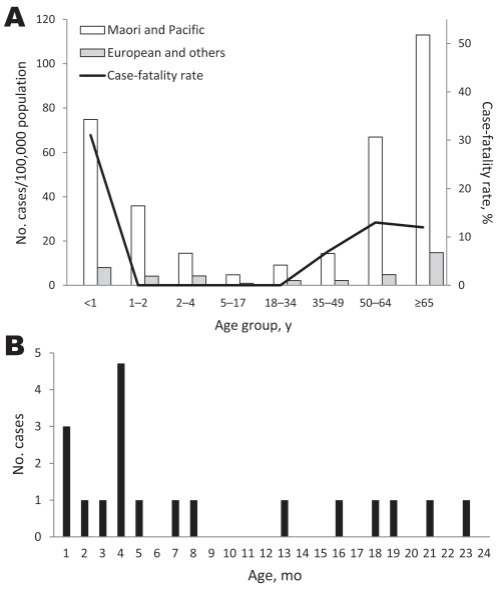
A) Annual incidence rates for invasive group A streptococcal (GAS) disease, Auckland, New Zealand, 2005–2006. The black line indicates age-specific case-fatality rates for combined ethnicities. B) Number of invasive GAS cases among infants <24 months of age.

The 225 patients were from all ethnic groups: European (77 [34%] patients), Maori (69 [31%]), Pacific Islanders (70 [31%]), and other ethnicities (7 [3%]). For 2 patients, no information was available about ethnicity. For the 225 patients, median age was 53 years (range 0–97 years), and 119 (53%) patients were male. Ethnic disparities, although notable in the extremes of life, did not differ significantly by age (Figure 1; Table 1). The 198 patients with invasive GAS infection for whom SES information was available were more likely to originate in areas designated by the New Zealand Deprivation Index 2006 as lower SES areas than in higher SES areas (Table 2). Forty-nine percent of case-patients were from the lowest SES quintile.

**Table 1:** Population-based incidence of invasive group A streptococcal disease, by age, Auckland, New Zealand, 2005–2006*

Population	Age group, y													
	<1			<15			<50			>65			All ages	
	No.	Rate		No.	Rate		No.	Rate		No.	Rate		No.	Rate
Maori and Pacific Islander	11	20.3		27	13.0		74	80.1		30	113.0		139	20.4
Maori	8	40.9		14	14.1		33	82.5		15	146.8		69	21.6
Pacific Islander	3	16.4		13	12.0		41	78.2		15	91.8		70	19.3
Other	2	4.1		9	2.4		53	8.9		36	15.0		84	5.3
Total	13	33.0		36	6.1		127	18.4		66	24.4		225	8.1

*Rate/100,000 population. Table includes only populations at risk. Use of a Poisson regression model indicated no evidence of a difference in the effect of ethnicity on risk in different age groups. The incidence rate ratios for all ages of Maori compared with others was 7.60 (95% confidence interval [CI] 5.10–11.32) and for Pacific Islanders compared with others was 8.84 (95% CI 6.17–12.65). For male vs. female, the incidence rate ratio was 1.29 (95% CI 0.96–1.74).

**Table 2:** Invasive GAS infection and relation with socioeconomic status, Auckland, New Zealand, 2005–2006*

New Zealand Deprivation Index 2006†	No. (%) confirmed invasive GAS infections, n = 198
10	63 (32)
9	33 (17)
8	23 (12)
7	23 (12)
6	10 (5)
5	10 (5)
4	6 (3)
3	10 (5)
2	15 (8)
1	5 (3)

*Based on the 198 case-patients for whom socioeconomic status information was available. GAS, group A streptococcal. †New Zealand Ministry of Health, www.moh.govt.nz. 10, most deprived area; 1, least deprived area.

### Case-Fatality Rate

Twenty-two patients died, for an overall case-fatality rate (CFR) of 10% (Figure 1, Table 3). Fourteen of these patients died within 72 hours after hospital admission. Three infants (one 2 months of age and two 4 months of age) who died in the community had STSS. One death previously had been attributed to sudden infant death syndrome.

**Table 3:** Clinical syndromes and CFRs for 225 patients with invasive GAS disease, Auckland, New Zealand, 2005–2006*

Diagnosis†	Age group, y					All ages, no. (%),‡ N = 225	p value§
	0–14, n = 36			>15, n = 189			
	No.	CFR		No.	CFR		
Skin and soft tissue infection¶	11	0		86	8	97 (43)	0.14
Bacteremia only	7	38		31	19	38 (16)	0.63
STSS#**	6	67††		24	54	30 (13)	0.59
Bone infection	6	0		20	0	26 (12)	0.39
Pneumonia and other respiratory infection	12	13		12	0	24 (11)	0.0001
Necrotizing fasciitis**	1	0		19	15	20 (9)	0.21
Pelvic infection/peripartum‡‡	0	0		12	0	12 (5)	

*CFR, case-fatality rate; GAS, group A streptococcal; STSS, streptococcal toxic shock syndrome. †Patients may have had >1 diagnosis, with the exception of bacteremia without a source. Other conditions (not shown) included 4 upper airway infections 6 ear/nose/throat infections, 5 central nervous system infections, 4 cases of peritonitis, 3 urinary tract infections, and 2 hemodialysis vascular access infections. No deaths occurred in this group. ‡Overall CFR 10% (22/225). §p value calculated by using Fisher exact test, a test of difference between age groups. ¶Includes cellulitis (n = 79), cutaneous abscess, boil, lymphadenitis, myositis, bursitis, infected burn, infected scabies, and infected ulcer with evidence of documented bacteremia. #STSS confirmed and probable (n = 3). **Five patients had STSS and NF; 1/5 died (20% CFR). ††Three of 6 were community deaths in infants <1 y of age. ‡‡Includes pregnancy-related (n = 6) endometritis and infected products, urinary tract infection/chorioamnionitis, and wound problems.

The median age of patients who died was 62 years (range 2 months–86 years). Eighteen adults who died had multiple concurrent illnesses. The highest CFR (31%) was for infants (a total of 4 deaths in three 4-month-old infants and one 2-month-old infant); these were the only deaths among children <15 years of age.

All infants who died had GAS-positive blood cultures. One who died in the community also had GAS-positive cerebrospinal fluid. Three of the 4 deaths occurred in the community and are attributed to STSS. The illness of the fourth (hospitalized) infant also met the criteria for STSS. Bronchopneumonia was found at post-mortem examination in 2 infants (1 hospitalized, 1 in the community). Two of the infants who died in the community had additional pathogens isolated from postmortem blood cultures (*Staphylococcus aureus* in both cases and *Streptococcus pneumoniae* and viridans streptococci in 1 each) but no gram-negative organisms.

### Clinical Features

The most common clinical feature was skin and soft tissue infection (97/225; 43%) (Table 3). Of the 30 patients with STSS, 26 (87%) had an underlying condition before the onset of acute GAS disease. Median age at STSS occurrence was 57 years (range 2 months–86 years). Six cases occurred in children <5 years of age. Empyema (4 cases; p<0.0001) and brain abscess (2 cases; p = 0.0011) occurred more frequently in children <14 years of age than in adults. The incidence of bacteremia with no focus of infection was 1.4 cases per 100,000 persons per year overall, but 3.7 per 100,000 for children <5 years of age (n = 7).

Seven cases of GAS postpartum infection were recorded for women 15–49 years of age, for a rate of 0.16 cases per 1,000 live-born infants (Maori and Pacific Islander, 0.21 cases/1,000 live-born infants). No deaths occurred in this group. We also identified 3 premature neonates with invasive GAS disease unrelated to cases in adults; 1 infection was nosocomially acquired.

### Risk Factors

Of the 223 patients for whom data were available, 58 (26%) had no underlying condition or other risk factor, 114 (51%) had 1 or 2 risk factors, and 64 (28%) had >3 risk factors. In the >15-years age group, 67 (35%) had heart disease, 60 (32% [23 Maori, 25 Pacific Islanders]) had diabetes, and 21% had either renal disease (39 persons) or lung disease (40 persons). Cigarette smoking was the most common nondisease-related risk factor (56 [30%] of 189 persons >15 years of age).

### Microbiological Analysis and Potential Vaccine-Preventable Disease

GAS was most frequently isolated from peripheral blood cultures (184 [82%]). Other sources were surgical specimen (37 isolates), tissue specimen (18), joint aspirate (16), pus aspirate (12), catheter blood culture (6), peritoneal aspirate (2), cerebrospinal fluid (2), and postmortem blood (3).

**Figure 2:**
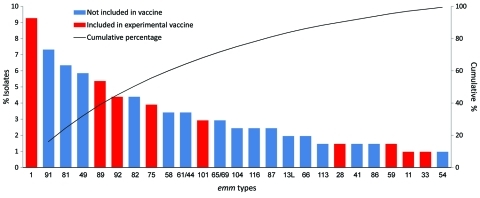
The 25 most common *emm* types as a proportion of all isolates. The remaining *emm* types were as follows: 100, 107, 25, 53, 56, 22, 18, 103, 105, 106, 108, 112, 123, 4, 51, 55, 70, 73, 77/27L, DRX4, ST6030, STN5554, 109, 110, 12, 52, 77, 88, 97, ST4119, ST4547, and 76.

Of the 225 cases, 205 (91%) GAS isolates were available for *emm* typing (Figure 2). Seventy (34%) of 205 cases had an *emm* type that was contained in the 26-valent vaccine. The proposed 26-valent vaccine could prevent 30% of GAS invasive cases in children <5 years of age and 15% of cases in persons >65 years of age (Table 4).

**Table 4:** Invasive GAS disease and fatalities potentially prevented by vaccination of infants and elderly persons with a proposed 26-valent vaccine, Auckland, New Zealand, 2005–2006*

Age group, y (no. *emm* typed)	Assumed vaccine efficacy, %	Assumed vaccine coverage, %	No. (%) persons with GAS disease from *emm* types in the 26-valent vaccine†	GAS-related deaths from emm type in the 26-valent vaccine†, %	Potential GAS disease prevented %‡	Potential GAS-related deaths prevented, %§
<5 (25)	>84	80¶	11 (44)	1#	29.6	0.67
>65 (59)	84	60**	18 (30.5)	14 (1/7)	15	7.1

*GAS, group A streptococcal. †Among patients with typed isolates. ‡Percentage of assumed vaccine efficacy × percentage of assumed vaccine coverage × persons with GAS disease from 26-valent *emm* types (based on O’Loughlin et al. [*2*]). §Percentage of assumed vaccine efficacy × percentage of assumed vaccine coverage × persons with GAS disease from 26-valent *emm* types × percentage of GAS-related deaths associated with a 26-valent *emm* type. ¶Craig et al. (*24*). #Of the 4 children <5 years of age, 2 had an *emm* typed isolate. Neither of these types is in the proposed vaccine. These are very small numbers. **New Zealand Ministry of Health Immunisation Handbook (www.moh.govt.nz).

Of the 225 isolates, 1 (0.4%) was resistant to erythromycin and 1 (0.4%) had intermediate sensitivity to erythromycin. Three (1.3%) were resistant to clindamycin.

### Disease Severity

Hospitalization was required for 222 patients (3 deaths occurred in the community). Length of stay was >10 days for 105 (47%); mean length of stay was 15.9 days (range 1–153 days). Thirty-eight (17%) required ICU admission (mean length of stay 4.5 days; range 1–9 days); maximum length of stay was 19 days. Nosocomial infection was responsible for 12 (5%) of the 225 cases. Seventy-five (33%) patients required at least 1 surgical procedure, predominantly drainage, débridement, or washouts. One patient (2 years of age) with STSS required intravenous immunoglobulin.

## Discussion

Our New Zealand study is population based and prospective. The overall annual incidence rate for greater Auckland of 8.1 cases per 100,000 persons per year is more than double or triple the rate of earlier reports elsewhere in the industrialized world. Annualized rates reported from other industrialized countries were 3.5 per 100,000 in 2007 in the United States (*2*); 1.5 in Ontario, Canada; and 3.1 in the Netherlands (*1**,**23*).

This study was conducted in metropolitan Auckland, where studies are ongoing to assess GAS disease, including endemic ARF (*11**,**12**,**24*), APSGN (http://dnmeds.otago.ac.nz/departments/womens/paediatrics/research/nzpsu/pdf/2008_report.pdf), and streptococcal pharyngitis (*25*) with associated *emm* typing. Our study was conducted in close association with ongoing active surveillance for ARF and its associated *emm* types (*26*) and APSGN surveillance. Our laboratory-based surveillance was supported by discharge data evaluation, chart review, and coroner surveillance, which minimized underestimation of STSS and NF. Approximately 50–70 new ARF cases (90% in persons <20 years) occur each year in this population (*12**,**24*) and a similar number of APSGN. The incidence of streptococcal pharyngitis has been carefully determined in a randomized controlled trial for ARF control at ≈60 cases per 100 child-years during a 4-year period in a population of ≈12,000 persons 5–19 years of age (*11*). This rate is considerably higher than that documented recently from Fiji (14.7/100 child-years) (*27*). Serotypes in ARF cases in our study were diverse (*emm* 58, 74, 75, 76, 92, 99, and 53), mirroring an earlier report (*emm* 53 and 58 associated with ARF) (*28*).

The annualized rate for Maori and Pacific Islanders <1 year of age (75/100,000) was similar to rates reported from Kenya (*29*) and greater than the rate more recently reported from Fiji (44.9/100,000) (*27*) from prospective studies. Nearly 50% of cases occurred in the lowest SES quintile of Auckland. Indigenous Maori and Pacific Islanders are overrepresented in lower SES areas of Auckland. Ethnically disparate rates for invasive GAS parallel these findings, with overrepresentation of these groups. The New Zealand Deprivation Index uses multiple parameters, including housing, income, and education. In addition, access to health care may be deficient (*30**,**31*) and perhaps health knowledge as well. The role of crowded housing in the population in our study has been recently documented for epidemic meningococcal disease (*32*) and may have a more substantial role for GAS disease, which is considered to be even more contagious (*33*). The high likelihood of an associated risk factor in the adult population, such as a chronic disease or another association, has been reported many times (*1**,**2**,**27**,**34*).

Skin infections have been documented as a major cause of illness in Auckland (*35*). More recently, New Zealand surveillance data (*24*) reported highly discrepant hospitalization rates for serious skin disease: Maori and Pacific Islanders <15 years of age are more likely to be hospitalized (unadjusted rate ratios 2.77 [95% CI 2.66–2.88] and 4.47 [95% CI 4.27–4.68], respectively) than are New Zealand European children 0–14 years of age. These data also reflected more hospitalizations for persons living in the most deprived quintile (*24*), which most likely is related to poor access to primary care and perhaps health knowledge. High population-based rates of invasive disease caused by methicillin-sensitive *S. aureus*, mostly bone and joint disease, also have been documented (*36*).

The overall CFR from our study (10%), with a high CFR for STSS (63%), mirrors other studies in the industrialized world (*2*). This CFR suggests good access to hospital care and efficiently delivered secondary and tertiary care, including ICU admission. A recently reported CFR (28%) from Fiji suggests otherwise from the developing world (*27*).

We included in our study all 3 infants who died in the community and from whom GAS was cultured (*37*). GAS is a rare finding from postmortem specimens (J. Zucollo, pers. comm.). In all 3 cases, only gram-positive organisms were isolated (1 solely group A streptococcus from blood and cerebrospinal fluid). Studies in which careful precautions have been taken to reduce contamination show that approximately two thirds of blood cultures yield negative results, 2 in 9 yield 1 isolate, and 1 in 9 show mixed growth. GAS infection as the sole cause of death was less certain in 2 cases in our study in which >1 potentially disease-causing species was cultured. We characterized the 3 infant deaths as STSS according to Davies et al. (*1*), a definition that produces higher rates of STSS and a higher CFR in children than in other reports. We look forward to further investigations in this area.

Current health strategies for preventing illness and death from invasive GAS infections are limited. The rate of nosocomial infection in our study was low. The high rates in postpartum women and in infants require further investigation. We were unaware of any links between cases in our series. In New Zealand, index cases of invasive GAS disease are not investigated by public health authorities (*38*). Primordial strategies, such as of the provision of less-crowded housing (*32*) and hand-washing education, need further consideration (*39*).

The currently available vaccine most advanced in clinical trials (*14*) comprises 26 *emm* types representing population-based, practice-based, and historical assessments from the United States (*14*). Its applicability to the population in our study might be less than ideal. Thirty-four percent of disease was caused by *emm* types in the proposed 26-valent vaccine. Data are accruing from other sites (79% *emm* coverage with the 26-valent vaccine in the United States, 69% in Europe, and 40% in Fiji) (*4**,**14**,**27*). Our data can contribute to a recent global estimate suggesting the current formulation of an experimental multivalent GAS vaccine may not be ideal in areas of most need (*40*). The effectiveness estimate in our study (Table 4) suggests that fewer than one third of invasive GAS cases in children <5 years of age and perhaps 15% of cases in persons >65 years of age could be prevented. This finding is of particular concern in a New Zealand population where other GAS-associated diseases cause a substantial amount of illness and death.

The rates in our study, driven largely by high rates in indigenous Maori and Pacific Islanders, are higher than those previously reported from industrialized countries and similar to reports from Fiji and Kenya. The rates suggest a need for more investigation and planned interventions in populations at highest risk. Our study also supports the role of GAS as a pathogen for invasive disease, particularly because of its effect on all age groups.
